# VEGF is upregulated by hypoxia-induced mitogenic factor via the PI-3K/Akt-NF-κB signaling pathway

**DOI:** 10.1186/1465-9921-7-37

**Published:** 2006-03-02

**Authors:** Qiangsong Tong, Liduan Zheng, Li Lin, Bo Li, Danming Wang, Chuanshu Huang, Dechun Li

**Affiliations:** 1 Department of Anesthesiology and Critical Care Medicine, Johns Hopkins University School of Medicine, Baltimore, MD 21287, USA; 2 Department of Medicine, Johns Hopkins University School of Medicine, Baltimore, MD 21287, USA; 3 Nelson Institute of Environmental Medicine, New York University School of Medicine, Tuxedo, NY 10987, USA; 4 Department of Pathology, Union Hospital of Tongji Medical College, Huazhong University of Science and Technology, Wuhan, Hubei 430022, China

## Abstract

**Background:**

Hypoxia-induced mitogenic factor (HIMF) is developmentally regulated and plays an important role in lung pathogenesis. We initially found that HIMF promotes vascular tubule formation in a matrigel plug model. In this study, we investigated the mechanisms which HIMF enhances expression of vascular endothelial growth factor (VEGF) in lung tissues and epithelial cells.

**Methods:**

Recombinant HIMF protein was intratracheally instilled into adult mouse lungs, VEGF expression was examined by immunohistochemical staining and Western blot. The promoter-luciferase reporter assay, RT-PCR, and Western blot were performed to examine the effects of HIMF on VEGF expression in mouse lung epithelial cell line MLE-12. The activation of NF-kappa B (NF-κB) and phosphorylation of Akt, IKK and IκBα were examined by luciferase assay and Western blot, respectively.

**Results:**

Intratracheal instillation of HIMF protein resulted in significant increase of VEGF, mainly localized to airway epithelial and alveolar type II cells. Deletion of NF-κB binding sites within VEGF promoter abolished HIMF-induced VEGF expression in MLE-12 cells, suggesting that activation of NF-κB is essential for VEGF upregulation induced by HIMF. Stimulation of lung epithelial cells by HIMF resulted in phosphorylation of IKK and IκBα, leading to activation of NF-κB. In addition, HIMF strongly induced Akt phosphorylation, and suppression of Akt activation by specific inhibitors and dominant negative mutants for PI-3K, and IKK or IκBα blocked HIMF-induced NF-κB activation and attenuated HIMF-induced VEGF production.

**Conclusion:**

These results suggest that HIMF enhances VEGF production in mouse lung epithelial cells in a PI-3K/Akt-NF-κB signaling pathway-dependent manner, and may play critical roles in pulmonary angiogenesis.

## Introduction

Vascular endothelial growth factor (VEGF), a dimeric 42-kd protein, is a multifunctional cytokine that plays a pivotal role in angiogenesis [1]. Expression of VEGF has been localized to perivascular cells in many organs, including the lung, and is critical for normal pulmonary vascular development [2]. Lacking even one allele of the VEGF gene leads to embryonic lethality with impaired vessel formation, and delayed endothelial cell development, and vessel sprouting, remodeling, and survival are also impaired [3,4]. VEGF is highly expressed by lung epithelial cells and plays an important role in maintenance of the differentiated state of blood vessels in pulmonary vascular beds [5,6]. VEGF acts mainly through two tyrosine kinase receptors, Flt-1 (VEGFR-1) and Flk-1 (VEGFR-2). Flk-1 is expressed in the vascular endothelium and is the earliest known marker for endothelium and endothelial precursors [7]. A null mutation in Flk-1 leads to the lack of a vasculature and results in very few endothelial cells, suggesting that Flk-1 functions in the differentiation and/or proliferation of endothelial cells [8]. In contrast, mice deficient of Flt-1 have excess endothelial cells that are not organized into normal tubular networks [9]. Since the importance of VEGF and its receptor in lung angiogenesis, development, and function maintenance, significant efforts have been made to elucidate the mechanisms that regulate their expression.

Hypoxia-induced mitogenic factor (HIMF) is a protein originally discovered in a mouse model of hypoxia-induced pulmonary hypertension [10]. Subsequent studies showed that HIMF is a lung-specific growth factor participating in lung cell proliferation and modulation of compensatory lung growth [10,11]. This cytokine-like factor possesses an angiogenic function that promotes vascular tubule formation in a matrigel plug model [10], and is developmentally regulated [12]. In addition, in cultured embryonic lung, HIMF exhibits antiapoptotic functions [12]. Our earlier studies have discovered that intratracheal instillation of recombinant HIMF protein induces widespread proliferation of airway epithelial cells, alveolar type II (ATII) cells, and cells in the lung parenchyma [11]. In this study, we further investigated the role of HIMF on VEGF expression in mouse lungs, and in cultured lung epithelial cells.

## Materials and methods

### Animal experiments

Adult male C57BL/6 mice (10–12 weeks old) were obtained from Jackson Laboratories (Bar Harbor, ME). Recombinant HIMF protein purification and HIMF intratracheal instillation were performed as previously reported [10,11]. All experiments followed the protocols approved by the Animal Care and Use Committee of Johns Hopkins University.

### Immunohistochemical staining for VEGF

Lung samples were processed and immunostained as previously described [10,12]. Polyclonal antibody for VEGF (1:200 dilution) was obtained from Santa Cruz Biotechnology (Santa Cruz, CA).

### Western blot for HIMF, VEGF, and GAPDH

Tissue collection, homogenization and protein electrophoresis were performed as previously described [11,12]. Protein (50 μg) or 40 μl of medium supernatant (for HIMF expression assay in cultured cells) from each sample was subjected to 4–20% pre-cast polyacrylamide gel electrophoresis (Bio-Rad, Hercules, CA). HIMF, VEGF, and GAPDH were detected with 1:1000, 1:500, and 1:1000 dilutions of antibodies, respectively, followed by 1:3000 dilution of goat anti-rabbit HRP-labeled antibody (Bio-Rad). ECL substrate kit (Amersham, Piscataway, NJ) was used for the chemiluminscent detection of the signals with autoradiography film (Amersham).

### Semi-quantitative RT-PCR for HIMF and VEGF

Total RNA was isolated with RNeasy Mini Kit (Qiagen Inc., Valencia, CA). The reverse transcription reactions were conducted with Transcriptor First Strand cDNA Synthesis Kit (Roche, Indianapolis, IN). The PCR primers were the following: for mouse HIMF 5'-ATGAAGACTACAACTTGTTCCC-3' (positions 104 to 125 of second exon) and 5'-TTAGGACAGT TGGCAGCAGCG-3' (positions 419 to 439 of fourth exon) amplifying a 336-bp fragment; for mouse VEGF 5'-TGGATGTCTACCAGCGAAGC-3' and 5'-ACAAGGCTCACAGTGATTT T-3' amplifying a 308-bp fragment between positions 522 and 829; for mouse GAPDH, 5'-GCCAAGGTCATCCATGACAACTTTGG-3' and 5'-GCCTGCTTCACCACCTTCTTGATG TC-3' amplifying a 314-bp fragment between positions 532 and 845. The ratios between the amplified DNA fragments and GAPDH for each sample RNA were quantified by Phoretix 1 D software (Phoretix International Ltd., Newcastle upon Tyne, UK).

### Cell culture and stimulation with HIMF

MLE-12 cells (ATCC, CRL 2110), an SV40-transformed mouse lung epithelial cell line of alveolar type II cell lineage, were grown in RPMI 1640 medium (Gibco, Grand Island, NY) containing 10% fetal bovine serum (FBS, Gibco), penicillin (100 U/ml) and streptomycin (100 μg/ml). After the cells reached 80–90% confluency, the cells were fed with a medium supplemented with 0.1% FBS and 2 mmol/L L-glutamine. Thirty-three hours later, cells were incubated in serum-free either RPMI 1640 for 4 h, and pretreated with LY294002, SB203580, PD98059 or U0126 (Calbiochem, La Jolla, CA) as indicated, then stimulated with different concentrations of HIMF protein for specified periods, with or without Actinomycin D (5 μg/ml, Sigma, St. Louis, MO).

### Establishment of stable HIMF overexpressing cell lines

Mouse HIMF cDNA was amplified from mouse lung tissue and subcloned into pcDNA3.1/Zeo (+) (Invitrogen, Carlsbad, CA). The primers used for the HIMF cDNA amplification were sense 5'-CACCATGAAGACTACAACTTGTTCCC-3' and antisense 5'-TTAGGACAGTTGGCAGCAGCG-3'. Dominant-negative mutants of IKKα [IKKα (K44A)], IKKβ [IKKβ (K44A)], IκBα super-repressor [IκBα (S32A/S36A)] and phosphatidylinositol 3-kinase (PI-3K) (Δp85) were previously described [13,14]. HIMF cDNA or dominant-negative mutants were transfected into MLE-12 cells with Lipofectamine 2000 (Life Technologies, Inc., Gaithersburg, MD). Stable cell lines, MLE-HIMF, and their transfection control (vector only) cells MLE-Zeo, were selected with Zeocin (400 μg/ml). HIMF expression was validated by Western blotting and RT-PCR analyses.

### Dual-luciferase reporter assay for VEGF and NF-κB

The mouse VEGF promoter-luciferase reporter constructs containing a series of deletion fragments from the 5'-flanking region of VEGF promoter, pGL-VEGF, pGL-VEGF (-HRE), pGL-VEGF (-AP1), pGL-VEGF (-AP2), pGL-VEGF (-NFκB) and pGL-VEGF (-SP1), were provided by Dr. S. Joseph Leibovich (Department of Cell Biology and Molecular Medicine, New Jersey Medical School, NJ) [15]. The NF-κB luciferase reporter construct pNFκB-Luc was purchased from Stratagene (La Jolla, CA). Cells were co-transfected with each reporter construct and the *renilla *luciferase vector pRL-TK (Promega, Madison, WI), with or without HIMF protein stimulation, and treated with passive lysis buffer according to the dual-luciferase assay manual (Promega). The luciferase activity was measured with a luminometer (Lumat LB9507, Berthold Tech., Bad Wildbad, Germany). The *firefly *luciferase signal was normalized to the *renilla *luciferase signal for each individual analysis.

### Phosphorylation Assay for IKK, IκBα and Akt

MLE-12 cells were treated with HIMF as described above. Protein (50 μg) from each sample was subjected to 4–20% pre-cast polyacrylamide gel (Bio-Rad) electrophoresis and transferred to nitrocellulose membranes (Bio-Rad), and then probed with rabbit anti-mouse antibodies against phospho-specific and non-phosphorylated IKK, IκBα and Akt (1:500 dilution, Santa Cruz Biotechnology), followed by 1:3000 dilution of goat anti-rabbit HRP-labeled antibody (Bio-Rad). ECL substrate kit (Amersham) was used for the chemiluminscent detection of the signals with autoradiography film (Amersham).

### Statistical analysis

Unless otherwise stated, all data were shown as mean ± standard error of the mean (SEM). Statistical significance (*P *< 0.05) was determined by *t *test or analysis of variance (ANOVA) followed by assessment of differences using SigmaStat 2.03 software (Jandel, Erkrath, Germany).

## Results

### HIMF enhances VEGF expression in mouse lungs

**Figure 1:**
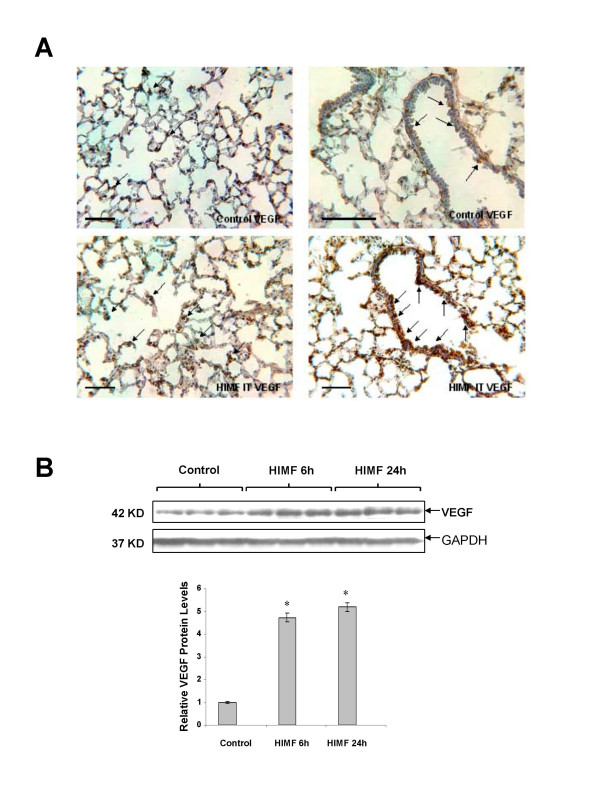
**HIMF enhances VEGF expression in mouse lungs**. Recombinant HIMF protein was intratracheally instilled into adult mice (200 ng/animal in 40 μl saline, n = 6 for each group). The vehicle controls were instilled with saline (40 μl/animal, n = 3). Six and twenty four hours later, the mouse lungs were collected. (A) The immunohistochemical staining results indicated that HIMF protein instillation resulted in a significant increase of VEGF production, mainly located at alveolar type II (left panels, arrows) and airway epithelial cells (right panels, arrows). Scale bars: 100 μm. (B) Western blot indicated that VEGF expression was enhanced in HIMF-instilled mouse lungs. The symbol (*) indicates a significant increase from control mouse lungs instilled with saline only (*P *< 0.05).

To examine the role of HIMF in VEGF expression in the lung, we intratracheally instilled recombinant HIMF protein into adult mouse lungs. We found that VEGF expression was significantly enhanced by HIMF stimulation, as demonstrated by immunohistochemical staining (Fig. 1A). The expressed VEGF was mainly localized within ATII and airway epithelial cells. Western blotting further confirmed the upregulation of VEGF in lung tissues after 6–24 h of HIMF-instillation (Fig. 1B).

### HIMF upregulates VEGF expression in mouse lung epithelial cells

**Figure 2:**
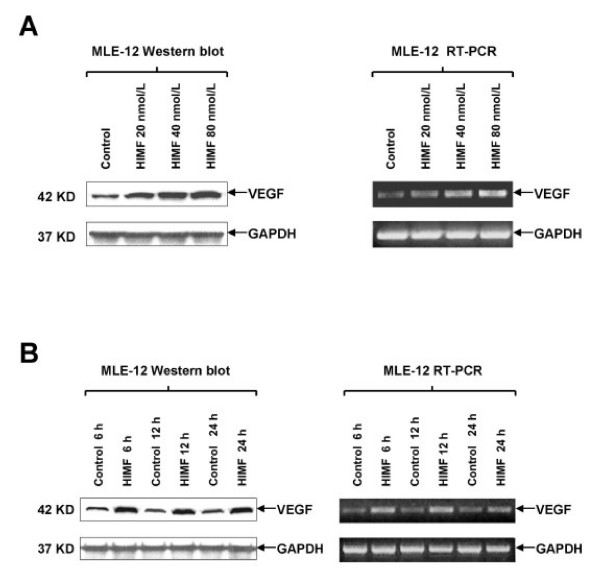
**HIMF induces VEGF expression in mouse lung epithelial cell line**. MLE-12 cells were treated with HIMF for various concentrations and periods as indicated. Western blots and semi-quantitative RT-PCR were performed for VEGF expression. (2A) HIMF induced VEGF protein and mRNA production in a dose-dependent manner in MLE-12 cells. (2B) Time-course study indicated that HIMF (40 nmol/L)-induced VEGF production started at 6 h, and persisted for 24 h. Triplicate experiments were performed with essentially identical results.

**Figure 3:**
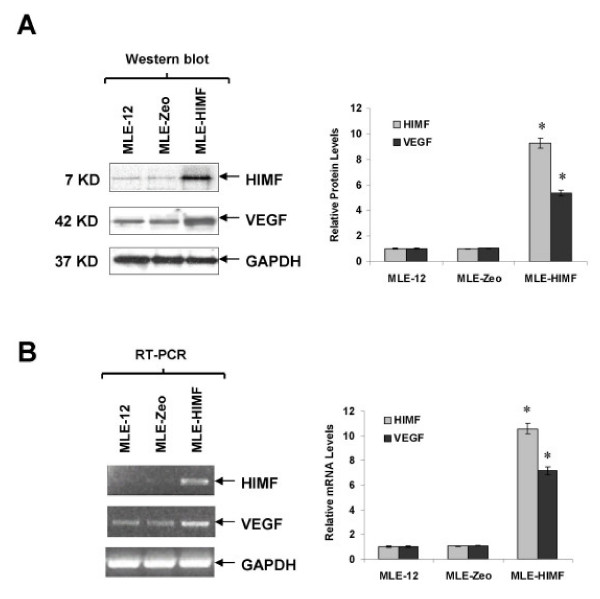
**Generation of HIMF overexpressing cells**. MLE-12 cells were transfected with HIMF cDNA or control vector. Stable cell lines, MLE-HIMF, along with their transfection control cells MLE-Zeo, were screened based on resistance to Zeocin (400 μg/ml). Western blots with cell culture medium for HIMF and protein from cell lysate for VEGF (3A) and RT-PCR with cell total RNA (3B) demonstrated that MLE-HIMF have higher HIMF protein and mRNA levels than their parent (MLE-12) and transfection (MLE-Zeo) counterparts. The VEGF levels in MLE-HIMF were also increased significantly compared with those of their controls. The symbol (*) indicates a significant increase from parent controls (*P *< 0.05). Triplicate experiments were performed with essentially identical results.

Although HIMF treatment leads to upregulation of VEGF, molecular mechanisms governing such induced expression in lung tissues remain unclear. To establish a cellular system for further investigating regulatory mechanisms of HIMF-induced VEGF production, we selected cultured MLE-12 (epithelial) cells as models. Western blotting of cell lysates showed that HIMF induces VEGF production in a dose-dependent manner in MLE-12 (Fig. 2A). These findings are consistent with the observations in lung tissues (Fig 1A). The induced expression of VEGF was further confirmed by RT-PCR in MLE-12 cells (Fig. 2A). Time-course studies showed that HIMF-induced VEGF production appeared at 6 h, and the expression level sustained for 24 h (Fig. 2B). VEGF also expresses to an elevated level in a cell line, MLE-HIMF that stably expresses HIMF (Fig. 3A and 3B). Successful recapitulation of HIMF-induced VEGF expression in epithelial cell line provides the basis for further dissecting the molecular mechanism of HIMF-induced upregulation of VEGF.

### HIMF modulates VEGF transcription, not its mRNA stability

**Figure 4:**
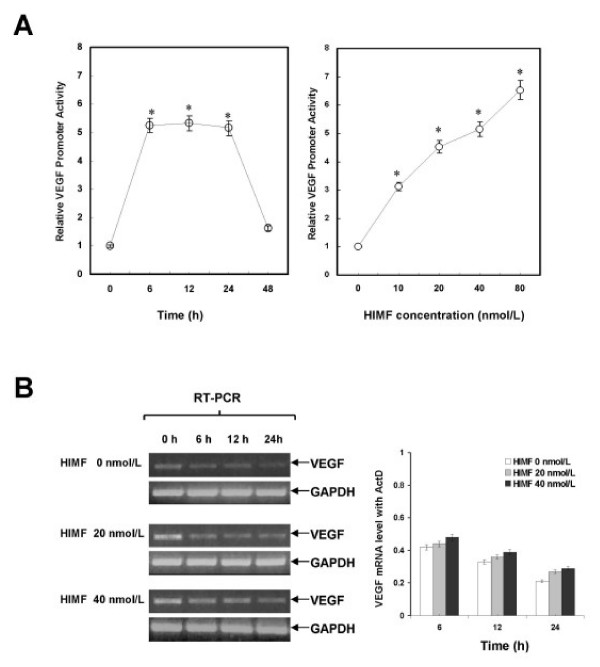
**HIMF increases the transcription activities, but not mRNA stability of VEGF in MLE-12 cells**. (4A) MLE-12 cells were co-transfected with pGL-VEGF and pRL-TK. Twenty-four hours later, the cells were incubated with HIMF protein as indicated. Then, cells were lysed with passive lysis buffer, and luciferase activity was measured according to the dual-luciferase assay manual. The time-course study demonstrated that HIMF-induced (40 nmol/L) VEGF transcription started at 6 h, and persisted for 24 h. After incubation with 10–80 nmol/L of HIMF, VEGF transcripts in MLE-12 were enhanced in a dose-dependent manner. (4B) MLE-12 were treated with different concentrations of HIMF and incubated with 5 μg/ml of Actinomycin D for 4, 8 and 16 h. RT-PCR indicated that HIMF did not prevent Actinomycin D-facilitated VEGF degradation in MLE-12 cells. The symbol (*) indicates a significant increase from MLE-12 controls without HIMF (*P *< 0.05). Triplicate experiments were performed with essentially identical results.

To test whether HIMF enhances VEGF expression at transcriptional level, we used a reporter construct, pGL-VEGF, which contains a luciferase gene driven by the VEGF promoter. The reporter plasmid was transiently transfected into MLE-12 cells and HIMF treatment of the transfected cells induced significant increases of luciferase activity in a dose-dependent manner (Fig 4A). It has been reported that VEGF mRNA stability is an important posttranscriptional parameter that modulates VEGF expression [16]. It is, therefore, possible that HIMF treatment enhances VEGF mRNA stability. To test this possibility, we used Actinomycin D, a transcription inhibitor that blocks transcription. However, VEGF mRNA degradation was still observed when treatment of MLE-12 cells with HIMF and Actinomycin D (Fig. 4B). These observations suggest that HIMF does not influence VEGF mRNA stability and the regulation of VEGF expression by HIMF is at transcriptional, rather than posttranscriptional level.

### Activation of NF-κB is essential for HIMF-induced VEGF expression

**Figure 5:**
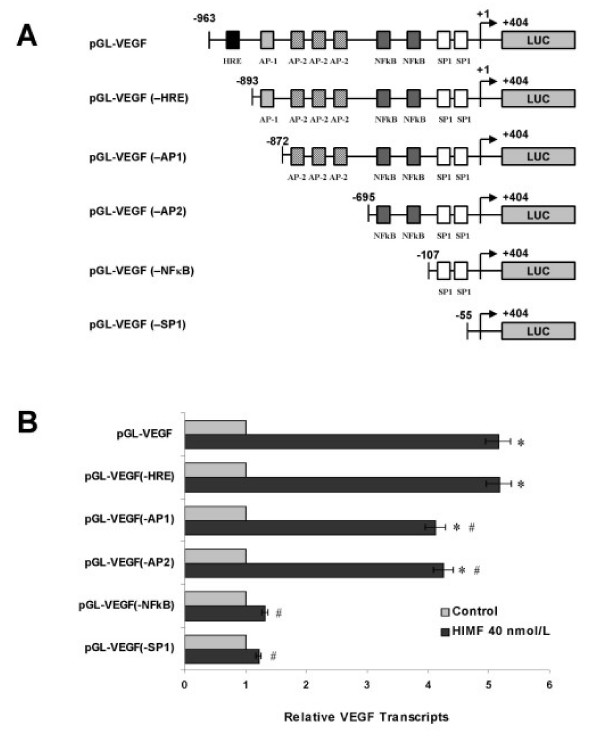
**Promoter deletion assay for HIMF-induced VEGF expression in MLE-12 cells**. MLE-12 cells were co-transfected with pRL-TK and each VEGF luciferase reporter construct (5A) for 24 h, then cells were incubated with HIMF protein (40 nmol/L) for another 24 h. Luciferase activity was measured and the *firefly *luciferase signal was normalized to the *renilla *luciferase signal for each individual well. (5B) Deletion of NF-κB binding site, but not HRE or binding sites for AP-1 and AP-2, completely abolished HIMF-induced VEGF promoter activity. Deletion of all *cis*-acting elements resulted in complete loss of induction of VEGF promoter activity. The symbol (*) indicates a significant increase from MLE-12 controls untreated with HIMF (*P *< 0.05). The symbol (#) indicates a significant decrease from MLE-12 transfected with pGL-VEGF and treated with HIMF (*P *< 0.05). Triplicate experiments were performed with essentially identical results.

**Figure 6:**
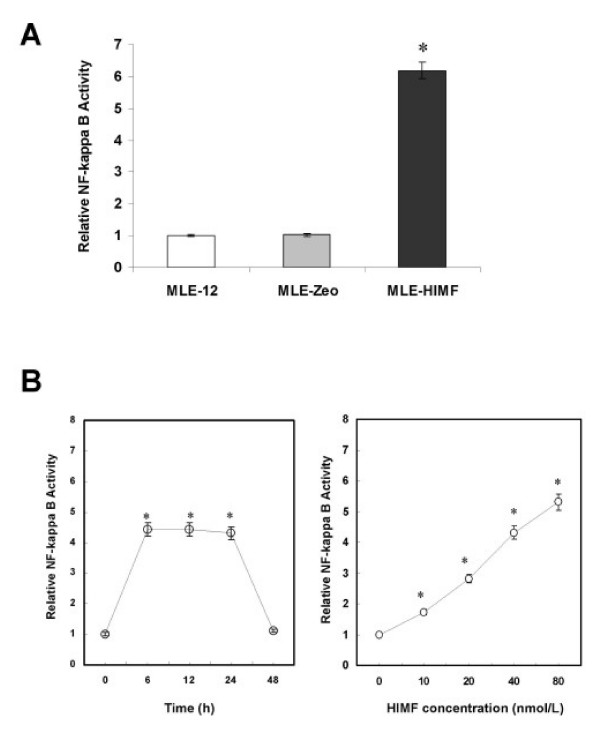
**HIMF activates NF-κB in MLE-12 cells**. Cells were co-transfected with pNFκB-luc and pRL-TK, with or without stimulation of HIMF protein for various periods as indicated. (6A) Dual-luciferase assay indicated that MLE-HIMF had higher NF-κB activity than their control counterparts. (6B) Dual-luciferase assay indicated that HIMF protein increased the NF-κB activity in MLE-12 cells in a dose-dependent manner. The symbol (*) indicates a significant increase from MLE-12 parent controls or controls untreated with HIMF (*P *< 0.05). Triplicate experiments were performed with essentially identical results.

After established that HIMF enhances VEGF expression at transcriptional level, we further explored the transcription factor(s) involved in the regulation. We used a series of luciferase reporter constructs containing different deletion segments of mouse VEGF promoter sequence [15], including hypoxia response element (HRE) and binding sites for AP-1, AP-2, NF-κB, and SP-1 (Fig. 5A). As shown in Fig. 5B, deletion of NF-κB binding site, but not HRE or binding sites for AP-1 and AP-2, completely abolished HIMF-induced VEGF promoter activity in MLE-12 cells. It has been reported that activation of NF-κB leads to the expression of VEGF [17]. We therefore tested whether HIMF induction would lead to activation of NF-κB, and subsequently, the expression of VEGF, using luciferase reporter assays. As shown in Fig. 6A, NF-κB activities in MLE-HIMF were significantly higher than those of their control counterparts. Consistent with the observation in MLE-HIMF cell line, incubation of MLE-12 cells with HIMF protein also induces NF-κB activity in a dose-dependent manner (Fig. 6B).

**Figure 7:**
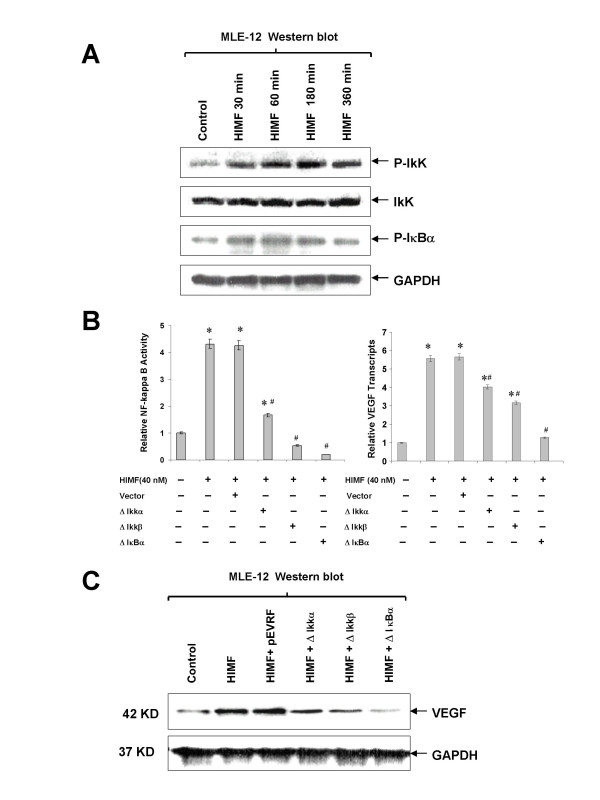
**Activation of NF-κB is essential for HIMF-induced VEGF expression**. Cells were co-transfected with pNFκB-luc, dominant-negative mutants of NF-κB pathway and pRL-TK, with or without stimulation of HIMF protein for various periods as indicated. (7A) Western blots indicated that HIMF (40 nmol/L) induced phosphorylation of IKK and IκBα in MLE-12 cells. (7B and 7C) Transfection of MLE-12 cells with dominant-negative mutants IKKα (K44A) and IKKβ (K44A), and super-repressor IκBα (S32A/S36A), abolished HIMF (40 nmol/L)-induced NF-κB activity and upregulation of VEGF in MLE-12 cells. The symbol (*) indicates a significant increase from MLE-12 controls untreated with HIMF (*P *< 0.05). The symbol (#) indicates a significant decrease from MLE-12 cells treated with HIMF only (*P *< 0.05). Triplicate experiments were performed with essentially identical results.

The prerequisite of NF-κB activation is the signal-dependent activation of the IKK-signalsome that contains IKKα and β kinases and other regulatory components. The IKK subsequently phosphorylates the inhibitor of NF-κB, IκB (IκBα as the dominant form in majority of cells). The phosphorylated IκBα is then degraded by the proteasome and releases bound NF-κB into the nucleus, leading to κB promoter/enhancer-specific gene expression. We found that HIMF induces phosphorylation of IKK and IκBα in MLE-12 cells (Fig. 7A), suggesting that HIMF signal goes through NF-κB route. Transfection of dominant negative mutants of IKK kinases, IKKα (K44A) and IKKβ (K44A), and an IκBα super-repressor, IκBα (S32A/S36A), abolished HIMF-induced NF-κB activity and production of VEGF in MLE-12 cells (Fig. 7B and 7C). Together, these findings demonstrated that activation of transcription factor NF-κB is essential for HIMF-induced VEGF production.

### PI-3K/Akt pathway is involved in HIMF-induced NF-κB activation and production of VEGF

**Figure 8:**
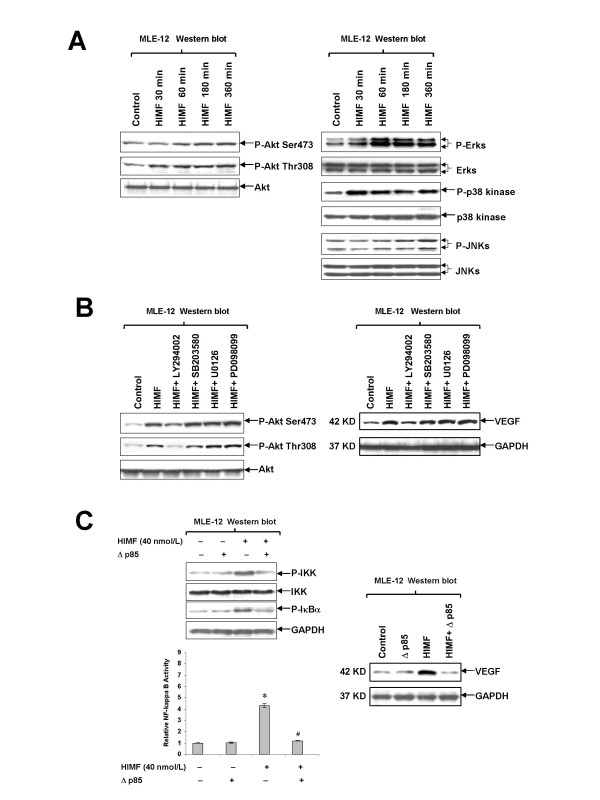
**HIMF-induced NF-κB activation and upregulation of VEGF is PI-3K/Akt pathway dependent**. MLE-12 cells were pretreated with signal transduction inhibitors or co-transfected with luciferase constructs and PI-3K dominant-negative mutant, then stimulated with HIMF (40 nmol/L) for various periods as indicated. (8A) HIMF strongly induced the phosphorylation of Akt at Ser473 and Thr308. The Akt phosphorylation started at 30 minutes and sustained for 360 min. HIMF also induced phosphorylation of ERK1/2 and p38 MAPK, but not JNK MAPK in MLE-12 cells. (8B) The PI-3K inhibitor LY294002 (10 μmol/L), but not SB203580 (5 μmol/L), PD098059 (5 μmol/L) or U0126 (5 μmol/L), abolished HIMF-induced Akt phosphorylation and upregulation of VEGF in MLE-12 cells. (8C) Transfection of Δp85, into MLE-12 cells abolished HIMF-induced phosphorylation of IKK and IκBα, NF-κB activation and production of VEGF. The symbol (*) indicates a significant increase from MLE-12 controls without HIMF treatment (*P *< 0.05). The symbol (#) indicates a significant decrease from MLE-12 cells treated with HIMF only (*P *< 0.05). Triplicate experiments were performed with essentially identical results.

It has been reported that HIMF also activates PI-3K/Akt signaling pathway. It is unclear, though, whether there are interplays between PI-3K/Akt and NF-κB pathways, and whether such interplays are necessary for HIMF-induced VEGF production. We therefore first tested the activation of main components of PI-3K/Akt signaling pathway upon HIMF treatment by Western blotting. As shown in Fig. 8A, HIMF strongly induces the phosphorylation of Akt at Ser473 and Thr308, ERK1/2 and p38 MAPK, but not JNK MAPK in MLE-12 cells. The Akt activation appeared at 30 min upon HIMF treatment, and sustained till 360 min. The PI-3K inhibitor LY294002 suppressed HIMF-induced Akt phosphorylation and upregulation of VEGF (Fig. 8B). Inhibitors to p38 and ERK1/2 MAPK pathways, SB203580, PD098059 or U0126, respectively, did not block Akt phosphrylation and VEGF expression induced by HIMF (Fig. 8B). Further, we found that transfection of Δp85, a dominant-negative mutant of PI-3K, into MLE-12 cells abolished HIMF-induced phosphorylation of IKK and IκBα (Fig. 8C), suggesting that PI-3K signaling acts at upstream of IKK signalsome. Consisting with this notion, Δp85 blocked HIMF-induced NF-κB activation as demonstrated by reduced NF-κB luciferase activity, and the production of VEGF (Figs. 8C). Together, our studies suggest that interplays between PI-3K/Akt and NF-κB signaling pathways are essential for HIMF-induced VEGF expression in lung epithelial cells.

## Discussion

A role for VEGF signaling in lung vascular development is suggested by the expression pattern of VEGF and its receptors [18]. Epithelial cells express VEGF during the process of lung morphogenesis, and the expression of its receptors is found in mesenchymal cells [18,19]. The complementary expression of this ligand-receptor system suggests the involvement of VEGF in the regulatory interactions between epithelial and vascular cells during lung morphogenesis [18]. Our previous study showed that HIMF is not expressed in the early pseudoglandular stage but begins at the terminal sac and alveolar stages. HIMF expression continues to be markedly upregulated until postnatal day 30 [12]. The temporal and spatial expression of HIMF in the perinatal period suggests its important roles as a coordinator of epithelial and endothelial growth during lung perinatal development. In the present study, we found that HIMF enhances VEGF production in mouse lung tissues and epithelial cell line. In contrast, Flk-1 expresses in endothelial cells, which can also be induced by HIMF via the PI-3K/Akt and NF-κB signaling pathways (preliminary studies, data not shown). Interestingly, HIMF-enhanced expression of VEGF occurs in epithelial cells (Fig. 2 and Fig. 6E), whereas Flk-1 induction occurs in endothelial cells (data not shown), suggesting that additional transcription factor(s) differentially regulates the cell type-specific expression VEGF and its receptor Flk-1. Identification of these transcription factor(s) should contribute significantly to our understanding of lung development and pathogenesis and certainly warrant further study.

Up to now, the organization of regulatory elements required for the expression of VEGF and Flk-1 has only been partially defined [15,17]. Several *cis*-acting elements, including the hypoxia response element (HRE) and binding sites for transcription factors, including AP-1, AP-2, NF-κB, and SP-1, are present in the murine VEGF promoter [15]. These elements are involved in the transcriptional activation of VEGF gene expression by numerous effectors, including hypoxia, growth factors and cytokines, such as TGF-α, TGF-β, IL-1β, and IL-6 [17]. Analysis of these elements revealed that HRE is required as a cis-element for transcriptional activation of VEGF by either hypoxia or nitric oxide [15,20]. Transcription factor AP-1 contributes to activation and expression of VEGF gene in hypoxic conditions. However, AP-1 is not necessary in the induction of VEGF gene expression [21]. NF-κB is involved in the upregulation of VEGF and its activity is associated with the high expression of VEGF mRNA [22]. In this current study, we found that HIMF protein enhances VEGF expression by inducing transcription factor NF-κB, that is required for VEGF promoter activity, rather than stabilizing VEGF mRNA posttranscriptionally.

In resting cells, NF-κB is sequestered in the cytoplasm through its interaction with the IκB (inhibitor of NF-κB) family of inhibitory proteins [23]. In response to external stimuli, IκB proteins undergo rapid phosphorylation on specific serine residues. Phosphorylation of IκBα on serines 32 and 36 and of IκBβ on serines 19 and 23 facilitate their ubiquitination on neighboring lysine residues, thereby targeting these proteins for rapid degradation by the proteosome [24]. Following the degradation of IκB, NF-κB is released and is free to translocate to the nucleus and to activate its cognate genes. A key regulatory step in this pathway is the activation of a high molecular weight IκB kinase (IKK) complex, termed IKK signalsome, in which catalysis is carried out by multiple kinases including IKKα and IKKβ [23]. Although NF-κB can be activated by different stimuli, the IKK signalsome serves as the key point that converges diverse upstream signals. In the present study, we found that phosphorylation of IKK and IκBα was induced by HIMF administration. Moreover, transfection of the dominant-negative mutants of IKKα and IKKβ, and an IκBα super-repressor abolished HIMF-induced NF-κB activation. These data support the notion that HIMF activates NF-κB through phosphorylation of IKK and IκBα.

Substantial progress has been made in understanding the signal transduction pathways regulating VEGF expression. Phosphatidylinositol 3-kinase (PI-3K) is a lipid kinase that is composed of two polypeptides, a p85 regulatory subunit, and a p110 catalytic subunit and is activated by a large spectrum of cytokines, hormones, and growth factors [25]. The serine-threonine kinase Akt is a downstream target of PI-3K [26]. Akt is regulated by phosphorylation at Thr308 and Ser473 residues by two phosphoinositide dependent protein kinases, PDK1 and PDK2 [26]. Activated PI-3K and Akt are strong inducers of neovascularization and endothelial cell proliferation [27]. VEGF levels are elevated in cells expressing activated PI-3K and Akt either by enhanced transcription or increased mRNA stability [27]. Our results demonstrated that HIMF induced Akt phosphorylation in MLE-12 cells. The time course of Akt phosphorylation is compatible with that of NF-κB activation in HIMF stimulated cells. Pretreatment of cells with LY294002, a PI-3K specific inhibitor, attenuated HIMF-induced Akt phosphorylation. Further, transfection of Δp85, blocked HIMF-induced phosphorylation of the IKK and IκBα, and hence NF-κB activation, and thus prevented upregulation of VEGF. These results suggest that PI-3K/Akt pathway is involved in HIMF-induced NF-κB activation and production of VEGF in mouse epithelial cells. Together, our studies identified a novel NF-κB activator, HIMF, and the upstream pathways that transduce HIMF signals from cell surface to nucleus, leading to the expression of VEGF.

In summary, our previous studies showed that HIMF possesses an angiogenic function that promotes vascular tubule formation in a matrigel plug model [10]. The current studies indicated that HIMF enhances VEGF production in mouse lung tissues and epithelial cells in a PI-3K/Akt-NF-κB signaling pathway-dependent manner, which at least in part, elucidated the molecular mechanisms of HIMF-elicited angiogenesis and contributed to a better understanding of the function of HIMF in lung angiogenesis and in the maintenance of pulmonary vascular homeostasis.
